# Atom–Photon Coupling from Nitrogen-vacancy Centres Embedded in Tellurite Microspheres

**DOI:** 10.1038/srep11486

**Published:** 2015-06-22

**Authors:** Yinlan Ruan, Brant C. Gibson, Desmond W. M. Lau, Andrew D. Greentree, Hong Ji, Heike Ebendorff-Heidepriem, Brett C. Johnson, Takeshi Ohshima, Tanya M. Monro

**Affiliations:** 1 ARC Centre of Excellence for Nanoscale BioPhotonics, Institute of Photonics and Advanced Sensing, The University of Adelaide, Adelaide, SA 5005, Australia; 2 ARC Centre of Excellence for Nanoscale BioPhotonics, School of Applied Sciences, RMIT University, Melbourne, VIC 3001, Australia; 3 Centre for Quantum Computing and Communication Technology, School of Physics, University of Melbourne, Parkville, VIC 3010, Australia; 4 Japan Atomic Energy Agency, Takasaki, Gunma 370-1292, Japan; 5 University of South Australia, Adelaide, SA, 5001, Australia

## Abstract

We have developed a technique for creating high quality tellurite microspheres with embedded nanodiamonds (NDs) containing nitrogen-vacancy (NV) centres. This hybrid method allows fluorescence of the NVs in the NDs to be directly, rather than evanescently, coupled to the whispering gallery modes of the tellurite microspheres at room temperature. As a demonstration of its sensing potential, shifting of the resonance peaks is also demonstrated by coating a sphere surface with a liquid layer. This new approach is a robust way of creating cavities for use in quantum and sensing applications.

As quantum information science seeks to move from laboratory-based proof of concept experiments into practical implementations, there is a need for robust and scalable quantum platforms^1^. The negatively-charged nitrogen-vacancy (NV) centre, consisting of a substitutionally nitrogen atom and an adjacent vacancy, shows great promise for quantum applications as it is robust, room-temperature active^2^, and relatively cheap when it is in the diamond (ND) form. However the integration of the NDs containing NV centres with cavity quantum electro-dynamic structures is still challenging^3^. Here we demonstrate a novel, robust and reproducible method to integrate NDs containing NV centres with a cavity by embedding NDs underneath the surface of the microspheres made of high refractive index tellurite glasses. We show the coupling of the emission from ensembles of NVs in the NDs to the Whispering gallery modes (WGMs) of the microspheres at room temperature. As a preliminary demonstration of the sensing potential, shifting of the resonance peaks is demonstrated by coating a sphere surface with a high refractive index liquid layer.

Since the report of single NV spin initialisation and readout^2^, the NV centre has become the most important solid-state, room-temperature compatible quantum platform. The ground state of the NV centre is an electronic spin triplet with a 2.88-GHz zero-field splitting between the magnetic sublevels^4^. With long coherence time, fast microwave manipulation, and optical preparation and detection, the NV electronic spin can be used to store quantum information and realize logic gates^5^. Spin dependent fluorescence intensity enables quantum sensing of magnetic^6^ and electric fields^7^, temperature^8^ and quantum fluctuations^9^.

To utilize NVs in quantum applications, efficient and scalable optical coupling between NVs and photonic devices including waveguides and cavities is advantageous. Cavities can play several roles in enhancing the operation of a NV-based sensor. They improve light collection, but perhaps more importantly, as the NV centre emission is extremely broad (>100 nm), Purcell enhancement can force the NV centre to emit in a narrow spectral window^10^,^11^,^12^,^13^, boosting the photon spectral density.

Reports to date of coupling between NVs and a cavity can be divided in two main categories: (i) monolithic all-diamond and (ii) hybrid approaches. All-diamond approaches are based on optical cavities fabricated directly in single crystal diamond substrates^3^,^14^. However, it is still challenging to grow and etch diamond single crystal thin films^12^. Hybrid cavities are typically fabricated in non-diamond materials and the fluorescence of the NVs is evanescently coupled to the cavities. Examples in planar platforms include 2D photonic crystal cavities in GaP substrates^15^,^16^, GaP microdisks on diamond substrate^17^, and 1D plasmonic cavity on silver nanowires^18^. Three dimensional polymer disk cavities with NDs mixed provided a platform for direct coupling of the NV emission into the resonance modes^19^. WGMs based on freestanding sphere cavities were also used for this purpose. Coupling between NV centres and silica^20^,^21^,^22^,^23^,^24^ or polystyrene^25^ microspheres has been demonstrated. In these hybrid sphere cavities, the NVs were either intrinsic to a subwavelength diamond substrate, which was positioned close to the spheres^20^, or within NDs which were coated or placed on the surface of the spheres^21^,^25^. In all cases, the emission of the NVs was coupled into the sphere cavities through the evanescent field of the WGMs. For the NVs embedded inside the subwavelength diamond substrate, the relatively large distance (order of tens of nanometres) between the NVs and the spheres resulted in a sub-optimal coupling efficiency^20^. The NDs placed on the surface by coating or nanomanipulation lead to additional scattering loss^21^,^25^. A further and even more significant drawback of this approach is the lack of mechanical stability of the NDs on the surface of the sphere, as the NDs can detach from the sphere, which leads to device failure.

To overcome this issue, we have developed a technique for creating high-Q tellurite microspheres with embedded NDs by simply heating the ND coated tellurite fibre tapers. This hybrid method allows the fluorescence emission of the NVs to be directly, rather than evanescently, coupled to the modes of the tellurite microspheres and creates an easy way to realize a robust cavity by embedding the NDs into the microspheres.

## Results

### Observation of the WGMs in the ND embedded tellurite spheres

**Figure 1:**
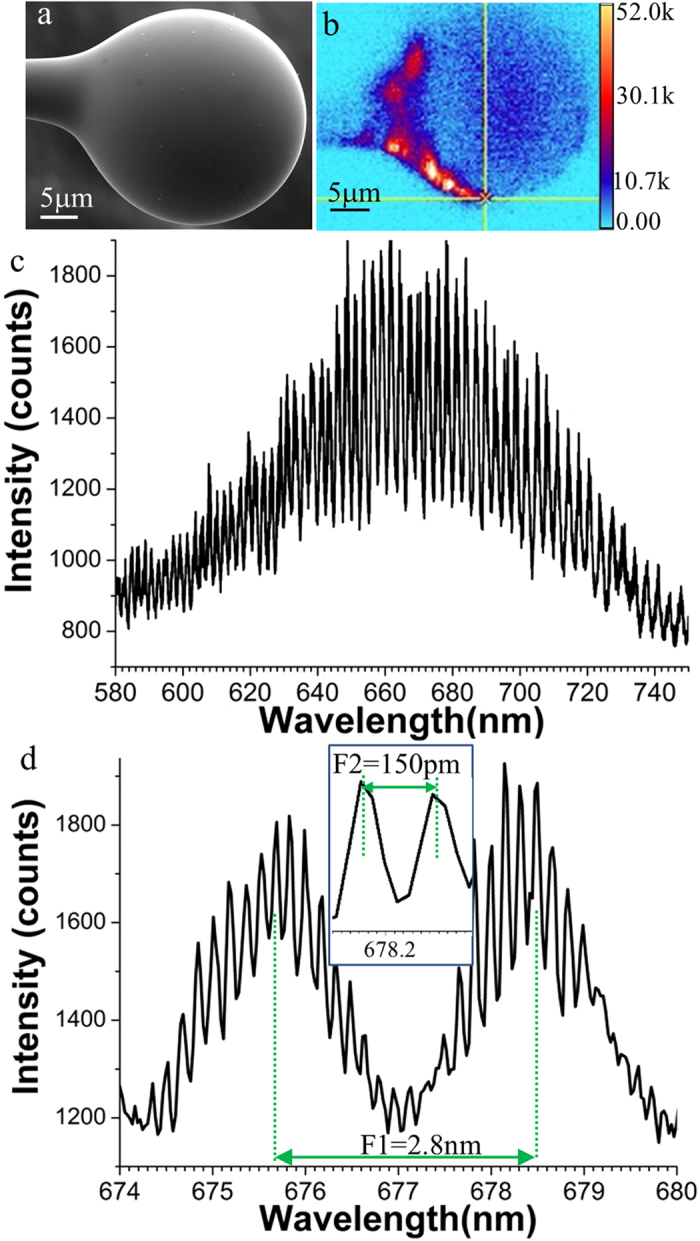
WGM excitation of one tellurite sphere embedded with NDs. (**a**) SEM and (**b**) scanning confocal images of a tellurite sphere with 28 μm diameter, respectively. (**c**) is the WGM modulated NV fluorescence when the spot marked on (**b**) was excited. (**d**) is a magnification of part of the spectra from (**c**), showing subpeaks of the main resonances. Also highlighted are the free spectral ranges of the WGM modes of F_1_ = 2.8 nm and F_2_ = 150 pm.

The scanning electron microscope (SEM) and scanning confocal images of one of our ND:tellurite spheres are shown in Fig. 1. We selected tellurite glasses as a host material for sphere fabrication since they are deformable at relatively low temperatures (400–700 °C). This temperature minimizes oxidation of the NDs while heating the glasses enabling fabrication of the spheres. Tellurite glasses transmit in the NV excitation and emission wavelength range of 500–800 nm, and have a high refractive index (n = 2.0), which better matches the refractive index of diamond (n = 2.4) than silica spheres (n = 1.45), and therefore reduces the scattering that occurs at the diamond/glass interface with ND:tellurite relative to ND:silica. Our previous work has shown coupling of the emission of the NVs into the ND doped tellurite fibres^26^,^27^,^28^. Here we demonstrate that the NV emission can also be directly coupled to the WGMs of the tellurite sphere cavities.

The ND:tellurite spheres were manufactured by dip coating tapered tellurite fibres into a solution containing NDs. The NDs were irradiated with a high-energy electron beam to increase their brightness^28^ (see Methods). The combined fibre and ND system was heated in a Vytran splicer to embed the NDs within the glass. For details, see Methods. Figure 1a shows an SEM image of one of the tellurite spheres made by this method.

Under scanning confocal imaging (Fig. 1b), several bright spots were observed on the surface of the microsphere. Figure 1c shows the spectrum obtained from the ND identified by the cross-hairs in Fig. 1b, showing NV emission with WGM modulation of the spectrum. Figure 1d is a magnified region of the spectrum in Fig. 1c. The spectrum was detected using a 1200i grating with 29 pm resolution. Figure 1d shows the presence of two competing WGM resonances (azimuthal and latitudinal modes) that were both coupled to the NV emission, indicating the sphere had a slightly nonspherical shape. The free spectral ranges of the resonances were *F*_1_ = 2.8 nm and *F*_2_ = 150 pm. We identified *F*_1_ with azimuthal modes while *F*_2_ corresponded to latitudinal modes^29^. *F*_1_ corresponds to a sphere diameter of 26 μm, which agrees with the measured value of 28 μm. *F*_2_ can be inferred from *F*_1_ by the ellipticity of the sphere, using *F*_2_ = *F*_1_(a-b)/b, where a and b are the semi-axes of the spheroid^29^. The inferred ellipticity, (a-b)/b, is approximately 5%, which is close to that obtained by measurement of the sphere from its SEM images in Fig. 1a (<9%).

A relatively high Q value of 10,400 was achieved for the excited latitudinal resonances, and several microspheres with similar Q values were fabricated. Q was determined from the measured spectra by Q = λ/δλ, where λ is the central wavelength of the resonance peak and δλ is relevant full width half maximum of the resonance peak. This Q value is of the same order as those achieved in Er-doped tellurite spheres (Q = 40,000)^30^. Reducing the surface density of NDs should minimise scattering from the ND and hence improve the Q of our microspheres further.

The NV spontaneous emission rate depends on local environment and can be enhanced by cavities. The enhancement can be quantified by the Purcell factor^3^


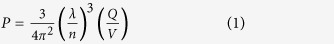


where λ is the wavelength and *V* is the mode volume. Note that this formula is only valid for an emitter located at the maximum of the electric field of the cavity mode with its dipole aligned with the local electric field^31^. In practice, the Purcell factor must be scaled down by 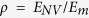, the relative strength of the electric field intensity at the NV location(E_NV_) compared to the mode maximum intensity (E_m_)^31^. The mode intensity distribution of the spheres can be solved by 2D finite element simulations^30^. For a 15 μm diameter tellurite sphere, our calculations show that the maximum electric field of the fundamental TM modes is located at the depth of 230 nm from the sphere surface. We can quantify the effect of depth on Purcell factor. For a 15 μm diameter tellurite sphere with a Q factor of 10,000, an NV centre just below the surface will have a Purcell factor of 0.43. Whereas, an NV centre at the electric field maximum will have a Purcell factor of 1.6. At present, we are unable to provide the exact depth of the NV centres. In addition, our microsphere-diamond system was in the bad-emitter regime, which has been explored recently in the context of semiconductor quantum dots in microcavities. In the bad-emitter regime, small mode volumes are necessary to show strong atom-photon coupling^32^.

### Q dependence on density of the embedded NDs

**Figure 2:**
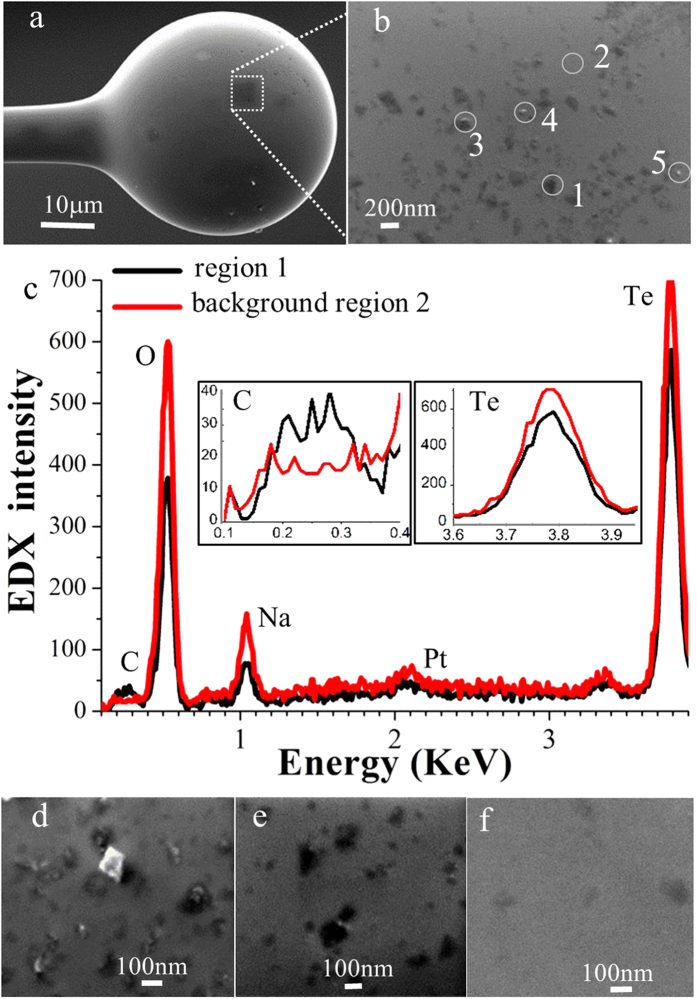
Q dependence on density of the embedded NDs. (**a**) SEM image of a tellurite sphere with 41 μm diameter and embedded NDs. (**b**) Magnified area of the sphere surface indicated by dashed lines. Five regions were identified as indicated. Element compositions of Regions 1 and 2 were analysed by EDX, and Regions 3 to 5 bright spots corresponding to unembedded NDs. Its measured ND surface density was 11/μm^2^. (**c**) EDX spectra showing the measured element compositions of region 1 and background region 2 in (b). (**d**)-(**f**) SEM images of magnified sphere surfaces for three other spheres with measured ND surface densities of 104/μm^2^, 14/μm^2^, and 3/μm^2^, respectively.

In Fig. 2a we show a SEM micrograph of a 41 μm diameter ND:tellurite microsphere, with a magnified region shown in Fig. 2b. Comparing these images to those of the tapers coated with unembedded NDs (not shown here), we can understand that the bright spots in Fig. 2b, for example Regions 3, 4 and 5, are resolved as unembedded NDs. The darker regions such as Region 1 arise due to NDs coated by the tellurite glass that is within the sampling depth of the SEM (in this case 100 nm for 20 KeV electron beam). Energy-dispersive X-ray spectroscopy (EDX) measurements of two regions from Fig. 2b are shown in Fig. 2c. Region 1 shows enhanced carbon signal and reduced signals of other glass elements compared to the background (Region 2), confirming the presence of the NDs.

Figure 2d–f show magnified surface areas for other three microsphere samples. The coated ND density (the number of NDs per unit area) on the taper tip prior to melting was determined by counting the NDs on the taper surface using their SEM images, and was 104/μm^2^, 14/μm^2^, and 3/μm^2^, respectively. As observed from their SEM images (Fig. 2d–f), after microsphere formation, some NDs were completely embedded under the surface with some of them only partly embedded. Our results indicate that lower ND density leads to a larger ratio of the embedded NDs compared with the total NDs on the sphere surface, and hence microspheres with higher Q. A dependence of Q on ND surface coverage was discovered for four spheres. Their Q values were not limited by radiation losses, which are negligible for tellurite spheres with diameter larger than 15 μm. Their ND densities were 0.06/μm^2^, 0.2/μm^2^ (sphere shown in Fig. 1), 5.16/μm^2^ and 7.16/μm^2^, respectively, with corresponding Q values of 10,000, 10,600, 5,000, and 7,000 respectively. This indicated a lower ND coating density led to a sphere with higher Q.

### Resonance shift with a coated liquid layer

**Figure 3:**
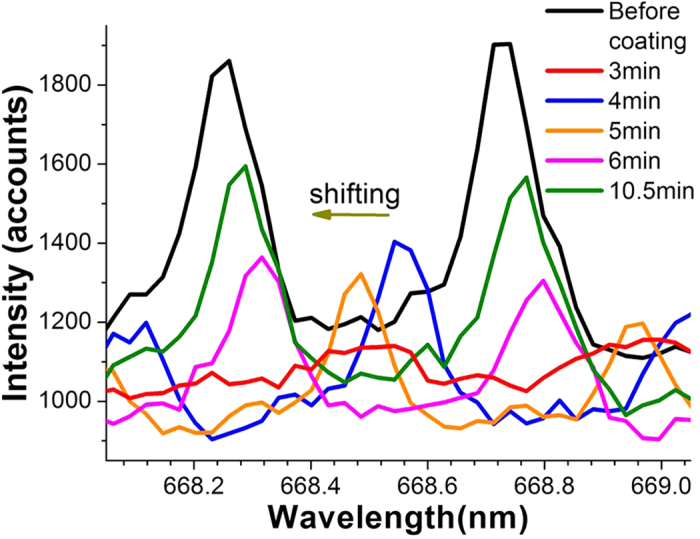
Monitored spectra as a function of time for a 17 μm diameter sphere coated with the liquid layer. The resonance intensity reduced after liquid coating. The intensity gradually increased as the resonance peaks shifted back toward to their original positions with time due to the liquid evaporating.

As a preliminary demonstration of the sensing potential, we also showed that the NV+WGM resonances could be shifted as shown in Fig. 3. This was achieved by applying a thin liquid film of index matching fluid (siloxane and aliphatic/alicyclic hydrocarbons with refractive index of 1.4 at 589 nm^33^) on the surface of a 17 μm diameter sphere with Q = 6,000 (linewidth = 110 pm) at λ ≈ 668 nm. Since the sphere diameter is ≈ 3 times larger than that of the taper region connected to the sphere, and the sphere was suspended in the air during optical characterisation, it was necessary to determine the effect of environmental fluctuations on the spectra. Prior to coating, we determined that the variation of the resonance peak positions within 20 mins was less than the resolution of the spectrometer. After the liquid layer was coated, the WGM spectrum was monitored for one hour to observe the peak position shift of the WGMs with time due to liquid evaporating.

Figure 3 shows the monitored resonance spectra after the liquid layer was formed. Noticeably, there was an initial drop in the fluorescence spectrum with the addition of the liquid, which can be identified with the liquid Q-spoiling the cavity. As the liquid evaporated, the WGM resonances re-established themselves at slightly red-shifted values, which relaxed to the original WGM spectrum and increased in contrast. These results are consistent with predictions of resonant Mie scattering theory for a fluid with refractive index lower than that of the microsphere^34^. We attribute the non-perfect recovery of the spectrum with un-evaporated residue from the index matching fluid.

## Discussions

In conclusion, we have demonstrated direct coupling of quantum NV emitters to high-Q high-index tellurite glass sphere cavities by embedding NDs underneath the sphere surface and optical excitation. These systems have the potential to be used as a robust and relatively simple platform for sensing and cavity-QED experiments.

For experiments at room temperature, the highest Purcell enhancement of all the spheres investigated here was calculated to be of 1.6 for a 15 μm diameter tellurite sphere with a measured Q factor of 10,000 when we assume the excited NVs located at the point with the maximum excitation electric field. Fabrication of tellurite spheres with smaller diameters, lower fractions of the embedded NDs and no contaminants should enhance the Purcell factor even further due to reduced mode volume and improved quality factors. Compared to other sphere cavities with the NV emission evanescently coupled to the resonance modes^20^,^21^,^22^,^23^,^24^,^25^, our tellurite spheres with NDs embedded have the potential to achieve the maximum Purcell factor if the NVs can be placed at the maximum mode electric field. For the same 15 μm diameter tellurite sphere with NDs embedded, the maximum Purcell factor is over 400 times larger than that of the same sphere with NDs located on the sphere surface, which could be realized by developing new processes to control the embedding depth of the NVs. For the spheres with NVs evanescently coupled to the WGM modes, their Purcell factor is always smaller than their maximum values^31^.

We also demonstrated a preliminary sensing geometry where a microsphere was immersed in index matching fluid, and the recovery of the resonances observed as a function of the evaporation of the fluid. The thickness of the liquid layers on the sphere surfaces was in the range of 2–12 nm, which was determined by observing the resonance shifts caused by these liquid layers.

Our experiments utilised high-brightness irradiation NDs. Whilst this helped signal to noise, it meant that we were unable to observe single photon emission from single colour centres. Future work will use these techniques to observe emission from isolated colour centres, and also correlate the effect of the dipole alignment of the emitter relative to the whispering gallery modes.

## Methods

### Fabrication of the ND doped tellurite spheres

TZN tellurite glass (TeO_2_-ZnO-Na_2_O) was used and fabricated in-house using the melt-quench technique in a gold crucible^35^. It has a glass transition temperature of 293 °C. The number of the gold ions dissolved from the crucible was minimised by using a melting temperature of 690 °C, which also avoided burning of the NDs^28^. The glass was extruded into a rod, which was drawn into an unstructured fibre with a diameter of 160 μm. The loss of the undoped tellurite fibres was measured to be 0.5 dB/m in the spectral range of 500–800 nm corresponding to the spectral range of NV absorption and emission^28^. The tellurite spheres were fabricated from the tellurite fibre using a Vytran splicer with an “Ω” shape iridium filament^30^.

We used 2 MeV electron-beam irradiated NDs^28^, which resulted in higher brightness than as-received commercial material. To obtain uniformly dispersed ND solutions for coating, the ND material (NaBond) was processed using strong acid reflux and ultrasonication^36^. The resulting supernatant in ethanol consisted of individual NDs with a mean size of 70 nm, and maximum size of 200 nm measured by dynamic light scattering. We used two different ND concentrations (0.1 and 0.5 mg/ml) for our experiments. The NDs were deposited onto the taper tips by time-controlled dip coating (less than 30 mins depending on the ND concentration). The fibre/taper length coated with the NDs was at least 2 cm.

### Experimental characterisation

Microspheres were imaged using SEM equipped with EDX for element analysis. The tapers and spheres were coated with a 3 nm thick platinum film for SEM imaging.

Our optical characterization system consisted of an in-house scanning fluorescence confocal microscope using a 100X objective (NA = 0.9) with low background fluorescence for excitation and signal collection, and diffraction-limited spatial resolution (~300 nm). A 532 nm diode laser was used for excitation of the NV centres within the individual NDs. The excitation power was 7 mW. The fluorescence signal was collected through a 532 nm notch filter and >560 nm long-pass filter and coupled into a multimode fibre for spectral analysis by a commercial spectrometer.

## Additional Information

**How to cite this article**: Ruan, Y. *et al.* Atom-Photon Coupling from Nitrogen-vacancy Centres Embedded in Tellurite Microspheres. *Sci. Rep.* **5**, 11486; doi: 10.1038/srep11486 (2015).
